# Not All That Is Droopy Post Ad26.COV2.S (JNJ) Vaccine Is Bell’s Palsy: A Rare Case of Isolated Dorsal Pontine Stroke Causing Ipsilateral Complete Hemi-Facial Palsy

**DOI:** 10.7759/cureus.23195

**Published:** 2022-03-15

**Authors:** Maryam Kundi, Sebastian Montgomery, Shirley Mao, Saleha Asghar

**Affiliations:** 1 Internal Medicine, Coliseum Medical Centers, Macon, USA; 2 Emergency Medicine, Coliseum Medical Centers, Macon, USA

**Keywords:** cva, stroke, janssen, jnj-78436735, bell’s palsy, vaccine adverse reaction, sars-cov-2, covid-19, ad26.cov2.s

## Abstract

The Ad26.COV2.S vaccine, developed by Janssen (Beerse, Belgium), the pharmaceutical wing of Johnson & Johnson (JNJ), is one of the three vaccines approved for use against coronavirus disease 2019 (COVID-19) infection in the United States. We present a case of a 66-year-old female who presented to the emergency department with a one-day history of nausea, vomiting, room-spinning vertigo, and complete right facial weakness immediately after getting vaccinated with Ad26.COV2.S. Initial workup focused on uncovering a possible association between the vaccine and Bell’s palsy. However, her prior history of stroke, presence of predisposing risk factors, and additional symptoms of nausea, vomiting, and vertigo prompted further neurological testing, which revealed an isolated right pontine lacunar infarct involving the right facial colliculus, mimicking Bell’s palsy. Isolated dorsal pontine lesion presenting as hemifacial palsy is very rare and can be easily missed by clinicians. Relevant history and thorough neurological examination can help guide appropriate diagnostic testing and prevent potential biases. It is crucial for clinicians to know the distinguishing features between true Bell’s palsy and acute brainstem infarction masquerading as Bell’s.

## Introduction

In the race to curb the spread of the coronavirus disease 2019 (COVID-19) pandemic, various vaccines were developed around the world. One such vaccine, the Ad26.COV2.S vaccine was developed by Janssen (Beerse, Belgium), the pharmaceutical wing of Johnson & Johnson (JNJ). This vaccine was built on Janssen’s AdVac® platform, which has been successfully utilized to create an Ebola vaccine regiment (Zabdeno® / Ad26.ZEBOV & Mvabea® MVA-BN-Filo) as well as in HIV, RSV, and Zika vaccine candidates. On February 27, 2021, the U.S. Food and Drug Administration (FDA) issued an Emergency Use Authorization (EUA) for the vaccine, allowing widespread use while additional data are gathered [1-2]. Data from the Phase 3 ENSEMBLE study [3], a randomized, placebo-controlled, double-blind study, showed 85% protection from severe COVID-19 disease beginning 28 days after vaccination. As a single-dose vaccine with prolonged stability in standard vaccine storage conditions, this vaccine has shown tremendous potential in the fight against COVID-19 and has been rolled out for use across the world. In clinical trials, there was concern regarding two cases of Bell’s palsy in vaccine wing trial participants, however, there were two notable cases of Bell’s palsy in the placebo wing trial as well. Clinical trial investigators concluded these events were unlikely to be related to the Ad26.COV2.S vaccine.

## Case presentation

**Figure 1:**
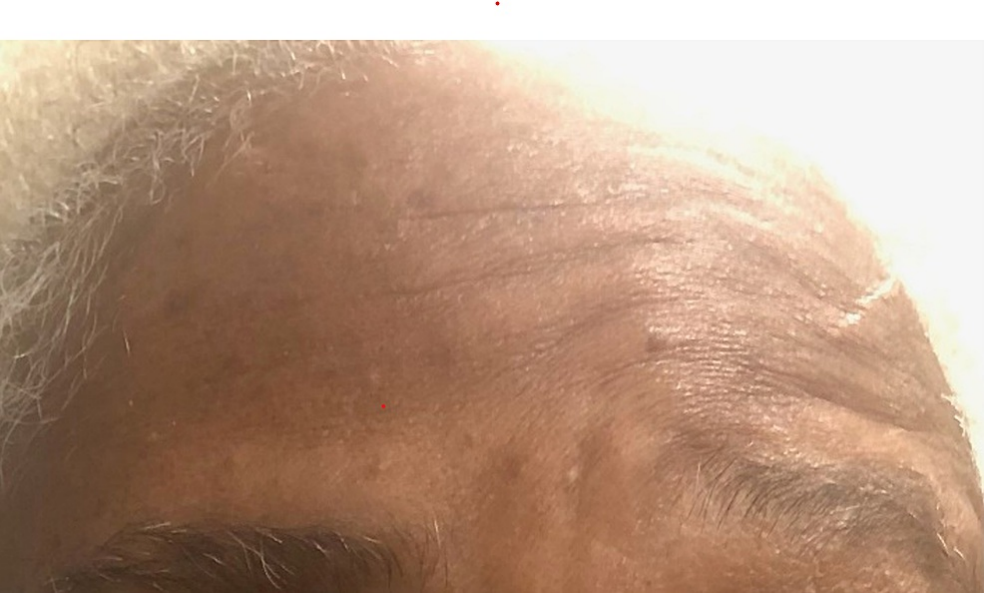
Loss of right forehead and eyebrow movement

**Figure 2:**
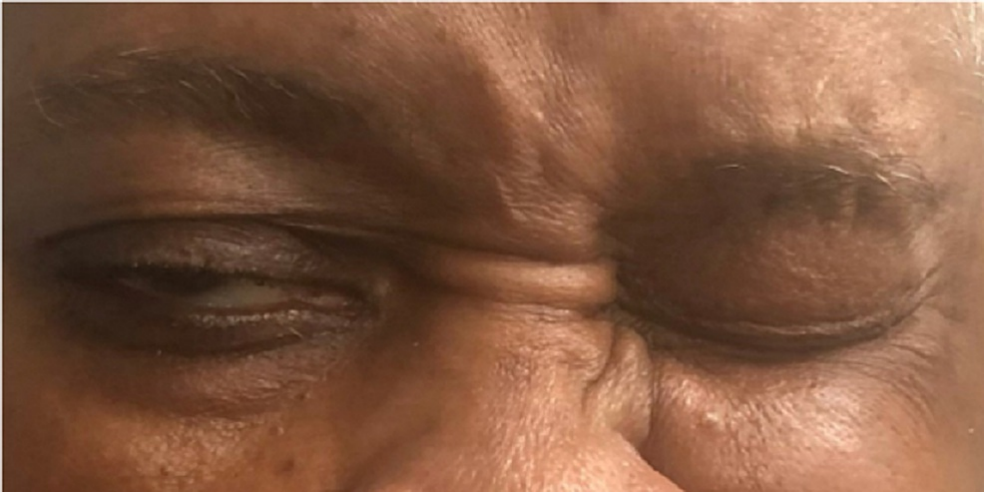
Inability to close right eye

**Figure 3:**
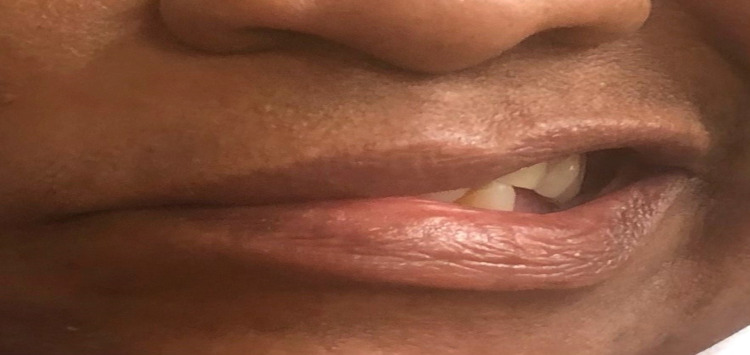
Loss of right nasolabial fold; asymmetrical smile with drooping at the corner of the mouth

A 66-year-old African American female with a relevant history of ischemic cerebral vascular accident (CVA) and without residual deficit, insulin-dependent diabetes mellitus type 2, and hypertension presented to the emergency department with complaints of nausea, vomiting, constant vertigo, and sudden right facial drooping progressively worsening over the past 24 hours directly following the first dose of Ad26.COV2.S vaccine. Additional history includes chronic anemia and depression. Her last hemoglobin A1c was unknown. She denied headache, vision or speech changes, trauma, or illicit drug use. She was taking aspirin 81 mg, pravastatin 10 mg, olmesartan or hydrochlorothiazide 40/25 mg, venlafaxine 150 mg, insulin lispro 10 units three times daily, and semaglutide 1 mg once a week at home. The patient reported compliance with her home medications. On presentation, the patient was alert and oriented with normal temperature and a pulse rate of 73 beats per minute. Her blood pressure was 187/79. The physical exam demonstrated Grade V right facial paralysis on the House-Brackmann scale. There was a loss of right forehead wrinkling (Figure 1) and an inability to close the right eye (Figure 2) and raise the right eyebrow (Figure 1), loss of nasolabial fold, and drooping of the corner of the mouth (Figure 3). No parotid swelling or masses were noted. Sensory and cerebellar exams were unrevealing.

Labs were significant for moderate anemia with hemoglobin of 7.7 and falsely low hemoglobin A1c of less than 3.8, which could be explained by her anemic state. Total cholesterol and low-density lipoprotein (LDL) were within normal limits, whereas high-density lipoprotein (HDL) was mildly elevated (Table 1). The patient was initially treated for possible Bell’s palsy following Ad26.COV2.S vaccine with prednisone 60 mg, valacyclovir 1000 mg, meclizine 25 mg, and ondansetron 4 mg.

**Table 1:** Laboratory Values * Indicates significant results HIV: Human Immunodeficiency Virus. HSV: Herpes Simplex Virus.

Laboratory Values	Result	Normal Range
Sodium	137 mmol/L	136-145 mmol/L
Potassium	3.5 mmol/L	3.5-5.1 mmol/L
Glucose	284 mg/dL *	74-106 mg/dL
Hemoglobin	7.7 gm/dL *	12-16 gm/dL
Platelet count	225 K/uL	140-440 K/uL
Total cholesterol	199 mg/dL	70-200 mg/dL
Low-density lipoprotein	108 mg/dL	0-130 mg/dL
High-density lipoprotein	79 mg/dL *	40-60 mg/dL
Lyme antibody	Negative	Negative
HIV 1, 2 antibody	Negative	Negative
HIV P-24 antigen	Negative	Negative
HSV 1, 2 DNA PCR	Negative	Negative
Prothrombin time/international normalized ratio	10.8 seconds/1.0	9.5-12.4 seconds/1.0
Activated partial thromboplastin time	22.2 seconds	22-34 seconds

**Figure 4:**
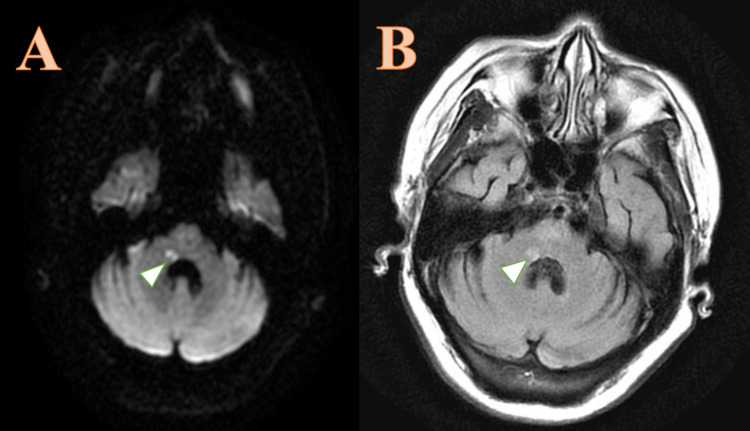
A) Axial diffusion-weighted imaging shows a tiny focus of diffusion restriction in the right pons on the floor of the fourth ventricle in the region of facial colliculus, shown by the arrowhead. (B) Axial T2 fluid-attenuated inversion recovery image (FLAIR) reveals a corresponding focus of hyperintensity signal

The electrocardiogram showed a normal sinus rhythm. Given the patient's chief complaint of persistent vertigo, CT head without contrast was performed in the emergency department for an immediate evaluation of cerebral infarction. This showed chronic right frontal and right parietal infarct with encephalomalacia consistent with old stroke and chronic microvascular ischemic changes. No acute large territorial infarct, hemorrhage, or masses were seen. Initially, the patient's lower motor neuron facial palsy seemed to suggest Bell's palsy, however, her additional physical exam findings and initial chief complaints and symptoms were more concerning for posterior circulatory ischemia, resulting in immediate CVA evaluation in the emergency department. Admission for further evaluation of ischemic stroke was required, as CT imaging alone would not rule out acute brain ischemia. CTA head with and without contrast exhibited multiple stenotic lesions of intracerebral arteries, indicative of widespread atherosclerosis. MRI of the brain without contrast revealed a tiny focus of diffusion restriction in the right pons and cerebellar peduncle with associated T2/fluid-attenuated inversion recovery (FLAIR) hyperintensity consistent with acute to subacute ischemia (Figure 4). Transthoracic echocardiogram (TTE) did not reveal any thrombi or structural heart disease. TTE, with a bubble study, and transesophageal echocardiogram (TEE) were not performed due to the low pretest probability of cardioembolic phenomena.

Management and outcomes

Steroid and antiviral treatment were stopped after MRI results revealed a lacunar pontine infarct. Dual antiplatelet and statin therapy were initiated. Blood pressure was medically optimized after a period of 24-48 hours to allow for permissive hypertension. The patient was discharged home after a three-day hospital stay on daily aspirin 81 mg, clopidogrel 75 mg, and atorvastatin 80 mg, and she was referred for outpatient follow-up with physical and occupational therapists.

## Discussion

Facial paralysis can be seen in many conditions, such as viral infections, pregnancy, autoimmune conditions, Lyme disease, stroke, and tumors. Idiopathic facial paralysis, also termed Bell’s palsy, is the most common cause of unilateral facial nerve palsy. Reports of Bell’s palsy post BNT162b2 (Pfizer-BioNTech, NYC, NY) and mRNA-1273 (Moderna, Cambridge, MA) have been published in several studies [4], however, a causal relationship between mRNA vaccines and Bell’s palsy is yet to be established. On the contrary, the incidence of Bell’s palsy post Ad26.COV2.S vaccine is rare. In clinical trials, the incidence of Bell’s palsy in Ad26.COV2.S vaccine recipients was the same as in the general population. The first case report of Bell’s palsy post Ad26.COV2.S was published on May 20, 2021 [5].

Our case presented on March 24, 2021. It was initially thought to be the first reported case of Bell’s palsy post Ad26.COV2.S but further testing (MRI studies) revealed an acute pontine stroke mimicking Bell’s palsy. Although Ad26.COV2.S has been linked to thrombosis with thrombocytopenia syndrome (TTS) in a case series [6], it was not the cause of stroke in our patient as many such cases presented with cerebral venous sinus thrombosis (CVST) with thrombocytopenia. Our patient had a normal platelet count and a normal coagulation profile. MRI did not demonstrate evidence of venous sinus thrombosis. Lack of thrombocytopenia did not prompt us to perform magnetic resonance venography (MRV). In addition, our patient also had a prior history of a right middle cerebral artery (MCA) ischemic stroke with predisposing risk factors; uncontrolled hypertension, and diabetes mellitus, hence it was challenging to establish a causal association between the vaccine and the event. The temporal relationship between the two was likely a mere coincidence.

Complete unilateral facial paralysis due to isolated dorsal pontine infarction is a very rare occurrence. Only 7% of all ischemic strokes are of pontine origin. Pontine lesions constitute only 1% of all new facial paralysis cases [7]. A review of the literature revealed only a handful of cases [8-9]. Nausea, vomiting, and vertigo were common occurrences in most cases similar to our patient, which can be explained by the involvement of closely located vestibulocochlear nerve nucleus in the brainstem. Hypertension and diabetes mellitus were also common risk factors. However, those cases did not present after Ad26.COV2.S vaccine in contrast to our patient but that could be no more than a chance association. It is important to differentiate between Bell’s palsy and ischemic stroke mimicking Bell’s palsy in the emergency settings as the management varies greatly. An acute ischemic stroke is a time-sensitive event, and early diagnosis is necessary to avoid delays in treatment. Pontine strokes are acute, usually accompanied by nausea, vomiting, numbness, dysarthria, dysphagia, diplopia, vertigo, or ataxia, whereas Bell’s palsy typically progresses over hours to days without associated symptoms [10]. Bell’s palsy is also more commonly seen in younger people.

Anatomically, upper motor lesions (central brain lesions) spare the upper facial musculature and affect the contralateral lower face because the forehead receives innervation from both the motor cortices. Conversely, lower motor lesions (peripheral lesions) affect all the ipsilateral facial muscles. The facial motor nucleus houses cell bodies of the facial nerve lower motor neuron (LMN) and is located in the facial colliculus of the dorsal pons. It is divided into a (1) Dorsal segment: Both motor cortices innervate this segment, which in turn supplies the ipsilateral upper face; and, a (2) ventral segment, which receives corticobulbar fibers from the contralateral motor cortex and innervates the ipsilateral lower facial muscles [11]. Ischemic insult to the facial colliculus in the dorsal pons will result in complete ipsilateral facial paralysis indistinguishable from Bell’s palsy as was seen in our patient.

## Conclusions

Ipsilateral complete facial paralysis due to isolated pontine lesions can be easily misdiagnosed as Bell’s palsy by clinicians due to the rare occurrence of the latter. It is crucial for clinicians to assess risk factors, perform a thorough neurological examination, and choose appropriate diagnostic tests before reaching a conclusion. In our case, the COVID-19 vaccination was a source of confounding bias, therefore, the patient’s symptoms were initially dismissed as Bell’s palsy but her prior history, predisposing risk factors, nausea, vomiting, and vertigo pointed toward a central etiology.
